# Rocuronium-Induced Rhabdomyolysis: A Case Report and Literature Review

**DOI:** 10.7759/cureus.94426

**Published:** 2025-10-12

**Authors:** Meidai Liang, Zhaoshuai Ji, Jing Tang, Yongfang Hu, Guangmeng Nie

**Affiliations:** 1 Department of Clinical Pharmacy, Beijing Tsinghua Changgung Hospital, School of Clinical Medicine, Tsinghua University, Beijing, CHN

**Keywords:** creatine kinase, drug-induced rhabdomyolysis, neuromuscular blocking agents, rhabdomyolysis, rocuronium

## Abstract

This case report explores the rare association between rocuronium, a non-depolarizing neuromuscular blocker, and rhabdomyolysis, a serious condition characterized by muscle breakdown. We present a case of an elderly patient who developed rhabdomyolysis after receiving intravenous rocuronium for airway spasticity and analgesic sedation. The patient exhibited a significant increase in creatine kinase (CK) levels, consistent with the diagnostic criteria for rhabdomyolysis. Our analysis indicates that rocuronium may have contributed to the development of this condition, especially with prolonged use and suboptimal anesthetic management. We recommend limiting the maintenance dose of rocuronium and closely monitoring patients for signs of muscle injury or elevated CK levels during and after treatment. This case highlights the importance of careful assessment and monitoring when using rocuronium, particularly in vulnerable populations, to mitigate the risk of rhabdomyolysis and other adverse effects.

## Introduction

Rhabdomyolysis is a serious condition characterized by the breakdown and necrosis of muscle tissue, resulting in the release of intracellular contents into the bloodstream [1]. The development of rhabdomyolysis is characterized by the release of large quantities of intracellular substances, including potassium, myoglobin, and creatine kinase (CK) into the circulation. This can lead to severe complications, such as intravascular volume depletion, metabolic acidosis, and multiple electrolyte disturbances, including hyperkalemia, hyperphosphatemia, and hypocalcemia, as well as acute kidney injury (AKI). The mortality rate for rhabdomyolysis is approximately 10%, and patients admitted to intensive care units (ICUs) are at higher risk, often due to underlying conditions such as trauma, sepsis, hypotension, electrolyte disturbances, and drug abuse [2,3]. Due to its diverse etiology, rhabdomyolysis is a significant concern in ICU patients with critical illnesses, who are particularly vulnerable to developing this condition [4]. Neuromuscular blocking agents (NMBAs) are commonly used to facilitate endotracheal intubation and maintain mechanical ventilation during prolonged procedures, thereby reducing ventilator dyssynchrony and respiratory workload, especially in patients with acute respiratory distress syndrome (ARDS). NMBAs can be broadly classified into two distinct categories: depolarizing and non-depolarizing agents. The depolarizing type, exemplified by succinylcholine, induces prolonged muscle depolarization and subsequent paralysis, characterized by muscle fasciculations and a phase I block that is resistant to reversal by acetylcholinesterase inhibitors. In contrast, non-depolarizing agents, such as rocuronium, function as competitive antagonists, blocking the binding of acetylcholine to its receptors and preventing depolarization, thereby causing muscle relaxation without eliciting fasciculations. Among NMBAs, succinylcholine has been implicated in cases of rhabdomyolysis. A non-diabetic, morbidly obese 32-year-old female patient developed rhabdomyolysis after receiving succinylcholine and sevoflurane for a 30-minute diagnostic procedure [5]. In contrast, rocuronium, a non-depolarizing neuromuscular junction blocker, is generally considered safer than succinylcholine and has been associated with a few reported cases of rhabdomyolysis. However, we present a case of rhabdomyolysis induced by rocuronium in a 74-year-old male patient and review the existing literature on this rare but potentially life-threatening complication.

## Case presentation

A 74-year-old male patient with a medical history of hypertension, chronic obstructive pulmonary disease (COPD), and hyperlipidemia presented to the emergency department with dyspnea following exposure to cold weather. Upon arrival, his vital signs were as follows: temperature 37°C, heart rate (HR) 136 beats per minute, respiratory rate (RR) 20 breaths per minute, and SpO₂ 92%. Laboratory findings revealed abnormal electrolyte levels, including sodium 134.1 mmol/L, chloride 94.6 mmol/L, and magnesium 1.06 mmol/L. Liver function tests were within normal limits, with aspartate aminotransferase (AST), alanine aminotransferase (ALT), lactate dehydrogenase (LDH: 216 U/L), and gamma-glutamyl transferase (GGT: 28 U/L) all within reference ranges. Urinalysis showed a pH of 5.5, trace occult blood (+/-), and no red blood cells (RBCs) on microscopy. Arterial blood gas analysis revealed type II respiratory failure with concomitant respiratory acidosis and metabolic acidosis, along with significantly elevated inflammatory markers. The clinical presentation was consistent with an acute exacerbation of COPD leading to type II respiratory failure.

**Figure 1:**
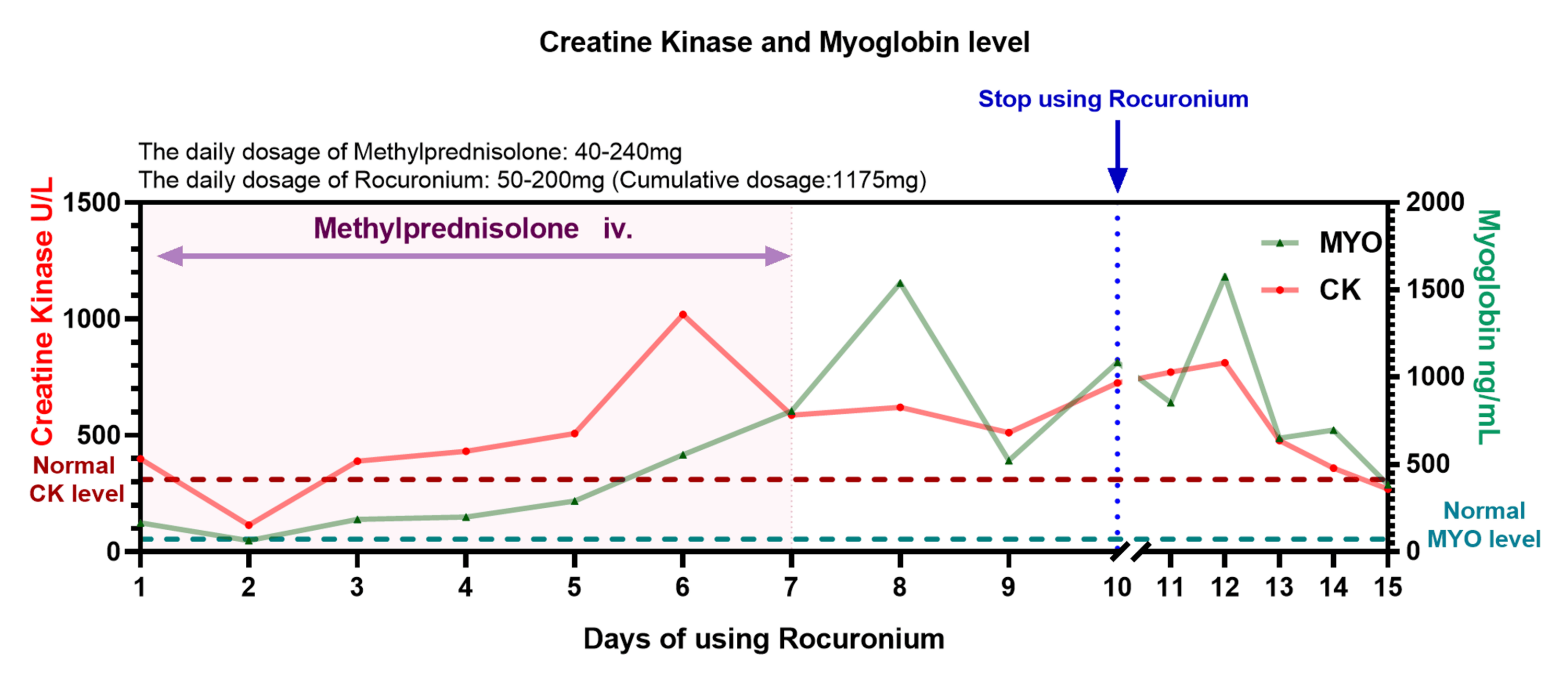
The patient's biochemical parameters, including CK and MYO levels during and after rocuronium administration Creatine kinase (CK) levels exhibited a significant elevation, rising from 399 U/L to a peak of 1,019 U/L on day 6 of rocuronium use, followed by a gradual decline to 512 U/L by day 9. Myoglobin (MYO) levels also showed a marked increase, from 165 to 1,539 ng/mL on day 8, with a subsequent reduction to 522 ng/mL by day 9. These trends are consistent with the development of rhabdomyolysis, a condition characterized by muscle breakdown and subsequent release of intracellular contents into the bloodstream

In response to the identified bronchial collapse in the right lower lobe, treatment strategies were optimized based on the findings from fiberoptic bronchoscopy. Sedation and NMBA were maintained, and tidal volume and the inspiratory-expiratory ratio were precisely adjusted to optimize mechanical ventilation. Additionally, bronchodilators and corticosteroids were administered to reduce airway inflammation and relieve bronchospasm (Figure 1).

However, during days 6-8 of rocuronium administration, the patient developed significant biochemical abnormalities, including elevated CK levels (from 399 to 1,019 U/L), myoglobin (from 165 to 1,539 ng/mL), and a rising plasma urea/creatinine ratio (from 0.15 to 0.35), as presented in Figure 1. Urinalysis consistently showed hematuria, progressing to 3+ on day 9 of rocuronium use, with a marked increase in RBCs to 250 cells/μL. The urine color darkened from pale yellow at admission to dark red and turbid, with microscopy examination revealing numerous RBCs and significant hematuria. Importantly, these abnormal laboratory and urinary findings resolved after the discontinuation of rocuronium. A review of biochemical data during rocuronium administration is summarized in Table 1.

**Table 1:** A review of biochemical data during rocuronium administration Rocuronium was continuously administered from 1 d to 10 d

Variable	1 d	2 d	3 d	4 d	5 d	6 d	7 d	8 d	9 d	10 d	11 d	Reference range
Urinalysis												
Blood in urine (cells/μL)	Trace (+/-)	-	-	-	-	-	-	-	250 (3+）	-	-	Negative (<25) cells/μL
Red blood cell (cells/HPF)	0	-	-	-	-	-	-	-	Full vision	-	-	0-3 cells/HPF
Color/turbidity	Yellow/clear	-	-	-	-	-	-	-	Red/turbidity	-	-	Yellow/clear
Blood												
Hemoglobin (g/L)	137	137	134	129	127	129	-	122	114	107	106	130-175 g/L
Platelet (×10^9^/L)	312	280	263	241	231	228	-	218	225	208	235	125 × 10^9^–350 × 10^9^/L
Biochemistry (plasma)												
Urea (mmol/L)	12.2	8.7	8.8	-	-	17.3	14.8	15.5	15.6	13.4	-	3.6-9.5 mmol/L
Creatinine (μmol/L)	79	67	49	-	-	50	46	52	47	53	-	57-111 μmol/L
Calcium (mmol/L)	2.01	2.03	2.05	-	-	2.01	1.98	1.97	1.91	2.05	-	2.11-2.52 mmol/L
Potassium (mmol/L)	3.77	3.85	3.37	-	-	3.84	3.77	4.06	4.24	3.7	-	3.5-5.3 mmol/L
Sodium (mmol/L)	139.1	146.7	148.5	-	-	148.3	151.2	149.7	147.7	148.6	-	137-147 mmol/L
Chlorine (mmol/L)	102.2	109.7	108.6	-	-	109.2	112.8	108.5	111.9	107.3	-	99-110 mmol/L
Urea/creatinine	0.15	0.13	0.18	-	-	0.35	0.32	0.3	0.33	0.25	-	-
Alanine aminotransferase (U/L)	23.4	21.6	17.7	-	-	14	-	-	17.4	-	-	9–50 U/L
Aspartate aminotransferase (U/L)	43.1	24.3	22	-	-	37.6	-	-	34.6	-	-	15–40 U/L
Creatine kinase (CK) (U/L)	399	114	389	432	508	1,019	587	620	512	725	772	50-310 U/L
CK MB isoenzyme (ng/mL)	13.3	5.34	3.8	1.92	1.75	3.77	2.35	2.22	1.63	2.76	3.72	0–6.22 ng/mL
Myoglobin (ng/mL)	165	63.4	185	199	291	556	806	1,539	522	1,085	854	28-72 ng/mL
Lactate dehydrogenase (U/L)	260	193	236	-	-	251	-	-	290	-	-	120-250 U/L
Gamma-glutamyl transferase (U/L)	100	84	68	-	-	58	-	-	63	-	-	15–65 U/L
Procalcitonin (ng/mL)	0.328	-	-	-	0.236	0.209	0.173	-	-	-	-	<0.05 ng/mL

The CK level normalized on the fifth day following drug discontinuation, while myoglobin levels returned to normal on the ninth day after cessation of the medication. The patient is still alive at the time of submission. After systematically excluding common causes of rhabdomyolysis, including trauma, epileptic seizures, and sepsis-induced muscle damage, our analysis suggests a potential association between rocuronium administration and the development of rhabdomyolysis.

## Discussion

The diagnosis of rhabdomyolysis is typically confirmed when the serum CK level exceeds 1,000 U/L or is at least five times the upper limit of normal [1]. While rhabdomyolysis can occur across all age groups, it predominantly affects adult populations, particularly those over 60 years of age. Obesity, along with electrolyte imbalances such as hyperkalemia and hypercalcemia, represents a significant risk factor that contributes to the development of this condition.

We encountered a case involving an elderly man with pulmonary infection and high airway resistance who was managed with intravenous midazolam, morphine, and rocuronium for airway spasticity and sedation. Continuous hydrocortisone sodium succinate was administered throughout his hospitalization. Over a six-day period of rocuronium treatment (cumulative dosage: 725 mg), the patient's CK level increased from 399 to 1,019 U/L, fulfilling the diagnostic criteria for rhabdomyolysis. After excluding common causes of rhabdomyolysis, such as trauma, epileptic seizures, and sepsis-induced muscle damage, we observed a significant increase in CK and myoglobin levels following rocuronium administration, suggesting a potential relationship between rocuronium use and the development of rhabdomyolysis.

Following the discontinuation of rocuronium, CK levels returned to baseline by the fifth day. A key clinical feature of rhabdomyolysis is muscle pain. However, in this case, the administration of analgesics may have masked this symptom. The first documented case of NMBA-induced myopathy in conjunction with steroid therapy dates back to 1977, involving a 24-year-old patient with asthma who received pancuronium and 3 g of hydrocortisone for 24 hours, resulting in quadriplegia [6,7]. However, the Committee on the Safety of Medicines raised concerns regarding the hypothesis that hydrocortisone was the primary cause of the myopathy, noting that the patient's myopathy was predominantly distal, whereas steroid-induced myopathy is typically characterized by a proximal distribution. Therefore, based on our findings, we suggest that the association between rhabdomyolysis and glucocorticoids is limited. Regarding the role of corticosteroids in rhabdomyolysis, Takahashi et al. reported a case where steroid treatment may have contributed to a rhabdomyolysis crisis in a patient with pheochromocytoma [8]. Conversely, there are also reports indicating that short-term high-dose corticosteroids can be an effective therapeutic option for recurrent rhabdomyolysis unresponsive to conventional fluid management [9]. These observations suggest that the effect of corticosteroids on rhabdomyolysis may be complex and dependent on individual patient factors and underlying clinical conditions.

Rocuronium is a non-depolarizing neuromuscular blocker with an intermediate duration of action and rapid onset, commonly used in general anesthesia to facilitate endotracheal intubation, mechanical ventilation, and various surgical procedures. Studies have demonstrated that rocuronium can be safely administered to critically ill patients under appropriate conditions without significant hemodynamic changes, rendering it a valuable option for minimizing interactions between patients and medical devices [10]. Rocuronium is currently considered a viable alternative to succinylcholine and is an ideal intubating muscle relaxant, producing acceptable intubating conditions within 60 seconds at doses of 0.9 and 1.2 mg/kg [11]. The relationship between NMBA use and the development of ICU-acquired weakness (ICU-AW) has been extensively studied, and it has been recognized as a risk factor for ICU-AW [12]. ICU-AW primarily comprises critical illness polyneuropathy (CIP) and critical illness myopathy (CIM). While manual muscle testing is a practical diagnostic tool for ICU-AW, electromyography (EMG) and nerve conduction studies (NCS) remain the gold standard for diagnosing this condition [13]. Rhabdomyolysis, characterized by fulminant and diffuse muscle damage, can be considered a subtype of ICU-AW, and in severe cases, CIM may progress to rhabdomyolysis [14]. Given the patient's prolonged deep sedation and coma, which precluded muscle strength testing, we diagnosed rhabdomyolysis based on elevated CK and myoglobin levels, rather than categorizing it as ICU-AW.

To date, only three documented cases of rhabdomyolysis attributed to rocuronium use have been reported in the literature (Table 2). Although the dosage of rocuronium was not recorded in the second case, it was postulated that the administration of high-dose methylprednisolone might have contributed to the development of rhabdomyolysis, suggesting a potential link between rocuronium and this adverse effect [15]. Crucially, the administered dose and duration of hydrocortisone sodium succinate in this case were within normal limits, which strengthens the hypothesis that rocuronium was the primary contributor to the observed rhabdomyolysis. We applied the Naranjo algorithm to evaluate the association between rocuronium and rhabdomyolysis. The final assessment score was 5. According to the scoring criteria, we conclude that rocuronium is a probable cause of rhabdomyolysis in this case.

**Table 2:** Reported cases of rhabdomyolysis with rocuronium administration CK: creatine kinase

Age, gender	Dosage of rocuronium	Onset time of rhabdomyolysis	Peak CK (U/L)	Combination therapy	Reference
36 years old, male	Route and dose not specified	On the fifth day after initiating methylprednisolone and after 7 days of rocuronium bromide administration	52,000	Methylprednisolone 1,000 mg/day and rocuronium bromide	[15]
37 years old, male	Route and dose not specified	About 10 hours after surgery	66,432	Propofol, vecuronium bromide, rocuronium bromide, and sevoflurane	[16]
8 months old, male	Dosages not stated	The cause of death of this infant is Duchenne’s muscular dystrophy (DMD) with rhabdomyolysis and an associated critical fever	Not stated	Died <24 h after receiving propofol, fentanyl, sevoflurane, and rocuronium anesthetic	[17]

A systematic review of existing literature was performed to evaluate the association between rocuronium administration and the development of rhabdomyolysis or elevated CK levels in patients undergoing tracheal intubation. A randomized, dose-finding phase II study found that CK levels were elevated in patients treated with 0.6 mg/kg rocuronium, despite the safe reversal of deep neuromuscular block by sugammadex [18]. Additionally, a study by Ren and Lv [19] investigated the neuromuscular effects of rocuronium and muscle injury in patients on long-term statin therapy who underwent general anesthesia with midazolam, etomidate, sufentanil, and rocuronium (0.9 mg/kg). The results indicated that myoglobin levels increased at four, 12, and 24 hours post-administration in the non-statin group, whereas CK levels began to rise at 12 hours and progressively increased up to 24 hours post-surgery, relative to baseline levels [19]. These findings suggest that, even in the absence of statin therapy, rocuronium may induce an increase in myoglobin and CK levels that may not manifest until several hours after administration.

We analyzed the potential mechanisms underlying rocuronium-induced rhabdomyolysis. The efficacy of neuromuscular blockade induced by rocuronium is modulated by alterations in respiratory pH, with a lower pH enhancing the block [20]. Consequently, respiratory acidosis induced by lung-protective ventilation may prolong the duration of neuromuscular blockade mediated by rocuronium [21]. Prolonged administration of rocuronium, particularly in ICU settings, has been associated with an extended half-life [22]. This prolonged exposure may facilitate the translocation of rocuronium across capillary membranes, potentially leading to direct neurotoxicity or functional denervation of muscle fibers [22]. Inadequate anesthetic management may impair oxygen delivery to tissues, including skeletal muscles, especially in patients with compromised circulation or respiratory support [23]. A hypoxic environment may thereby increase the susceptibility of muscle tissue to damage, potentially triggering rhabdomyolysis. Furthermore, physiological stress during general anesthesia may disrupt metabolic homeostasis and induce intracellular imbalances, which could indirectly contribute to or exacerbate the development of rhabdomyolysis.

In patients with ARDS, extended administration of NMBAs for up to 48 hours during the initial phase has been associated with sustained improvements in oxygenation parameters [24]. Based on a review of case reports and existing literature on rocuronium-induced rhabdomyolysis, we advocate for future studies or pharmacovigilance reports to confirm the association and refine dosing guidelines of rocuronium. This strategy is intended to mitigate the risk of rhabdomyolysis associated with its use.

## Conclusions

We report a case of rhabdomyolysis in an elderly patient who underwent intravenous administration of midazolam, morphine, and rocuronium for airway spasticity and analgesic sedation, along with continuous hydrocortisone sodium succinate infusion. Our analysis indicates that rocuronium was a significant contributing factor to the development of rhabdomyolysis in this patient. Patients receiving rocuronium are at risk of adverse reactions, including elevated CK levels and muscle injury. To mitigate this risk, we recommend limiting the maintenance dose of rocuronium to 0.1-0.2 mg/kg/dose for no longer than 48 hours and closely monitoring patients for signs of muscle injury or elevated CK levels during and after treatment.
